# Presence and Dominance of *Lactobacillus* in the Endometrial Microbiome and Age-Related Associations in Patients with Recurrent Reproductive Failure

**DOI:** 10.3390/diseases14060185

**Published:** 2026-05-22

**Authors:** Tatyana Bodurska, Tihomir Totev, Emiliana Konova

**Affiliations:** 1 Faculty of Medicine, Medical University—Pleven, 5800 Pleven, Bulgaria; t.totev@mail.bg (T.T.); eikonova@abv.bg (E.K.); 2 Clinical Institute for Reproductive Medicine—Pleven, 5800 Pleven, Bulgaria; 3 UMHAT “St. Marina”—Pleven, 5800 Pleven, Bulgaria

**Keywords:** *Lactobacillus*, uterine microbiome, recurrent reproductive failure

## Abstract

Objectives: To evaluate the presence and dominance of *Lactobacillus* in the endometrial microbiome and their age-related associations in a large group of Bulgarian patients with recurrent implantation failure (RIF) and recurrent pregnancy loss (RPL) who attend our clinic. Methods: This retrospective study included 199 patients (mean age: 35.69 ± 5.16) with RIF (*n* = 103) and RPL (*n* = 96) who visited our fertility clinic between October 2019 and November 2022. Endometrial samples were analyzed using real-time PCR for target DNA sequences. Results: Overall, 62.8% (*n* = 125) exhibited an absence of *Lactobacilli* in their endometrial samples, with 63.1% (*n* = 65) of the RIF group and 62.5% (*n* = 60) of the RPL group showing a lack of *Lactobacilli*, with no statistically significant difference between the groups (*p* = 0.926). A *Lactobacillus*-dominant microbiome was found in 23.6% of the entire cohort (*n* = 47), 25.2% of the RIF group (*n* = 26) and 21.9% of the RPL group (*n* = 21). A reduced abundance of *Lactobacilli* was identified in 13.5% of the cohort (*n* = 27), though to differing degrees. There was no significant relationship between the abundance of *Lactobacilli* and belonging to the RIF or RPL group. A statistically significant difference was found in the mean age of two groups in cases with a *Lactobacillus*-dominant microbiome (mean age of 36.4 ± 4.8 years in the RIF group and 32.5 ± 3.5 years in the RPL group) (*p* = 0.004). Conclusions: Our findings demonstrate a high prevalence of non-*Lactobacillus*-dominant microbiomes in a large group of Bulgarian patients with RIF and RPL and significant age-related *Lactobacillus* changes in the microbiome of patients with RPL. These results point to the potential role of the uterine microbiome and support the need for further prospective studies, especially in cases of advanced maternal age.

## 1. Introduction

According to current data, the uterus is not sterile and human fetal development is not a sterile process [1]. Bacterial cultures can be established from endometrial samples from asymptomatic fertile non-pregnant women, with *Lactobacilli* being the most abundant species [2]. The mucosal barrier is the first line of immune defense against the external environment. The presence of commensal microorganisms on the surface of the mucosa is in homeostasis with the host organism and prevents the colonization of pathogens [3]. The local microbiota may influence local innate and acquired immune responses, leading to a mild and balanced inflammatory milieu that is a favorable environment for implantation. In the case of *Lactobacillus* depletion and microbial colonization, many pathogenic mechanisms are activated, which may affect implantation and pregnancy outcomes. These mechanisms include altered endometrial expression of genes encoding for proteins involved in inflammatory responses, proliferation and apoptosis; abnormal expression of specific leucocyte subsets; abnormal infiltration of plasma cells; altered secretion of IgG, IgM and IgA antibodies; and increased interleukin release [4]. In *Lactobacillus*-dominant endometrial microbiomes, inflammation and pathologies are not present and *Lactobacillus* spp. are considered commensal species in the endometrium [5].

Age is a well-known factor that affects the abundance of *Lactobacillus* spp. in the female reproductive tract. Their abundance is stable until about 40 years of age [6]. In parous women over 36 years of age, intrauterine *Lactobacillus* abundance is significantly decreased, most likely due to disruptions to the cervical barrier [7].

The significance of a *Lactobacillus*-dominated endometrial microbiome for implantation and pregnancy outcomes has been established in multiple studies. Moreno et al. [8] evaluated the effect of non-*Lactobacillus*-dominated microbiomes in receptive endometria on pregnancy outcomes after embryo transfer. They found a significant decrease in implantation [60.7% vs. 23.1% (*p* = 0.02)], pregnancy [70.6% vs. 33.3% (*p* = 0.03)], ongoing pregnancy [58.8% vs. 13.3% (*p* = 0.02)] and live birth [58.8% vs. 6.7% (*p* = 0.002)] rates. This led to the emergence of the hypothesis that there are two types of monometric bacterial compositions: *Lactobacillus*-dominated microbiomes, where more than 90% of all isolated microorganisms are *Lactobacilli*, and non-*Lactobacillus*-dominated microbiomes, where *Lactobacilli* account for less than 90% [8].

Some authors do not support the idea that *Lactobacilli* have beneficial effects for implantation. The team of Keburiya [9] found that 89.2% of samples from patients with RIF had a microflora with a dominance of *Lactobacilli.* According to them, there is no clear evidence to support considering *Lactobacilli* abundance in the prognosis of pregnancy in cases with a *Lactobacillus*-dominated endometrial microbiome. Ichiyama et al. [10] also do not accept the concept of a *Lactobacillus*-dominated microbiome as a biomarker for implantation success. According to their study results, there was no difference in the number of RIF patients (51.2% ± 37.5%) without *Lactobacilli* in their endometrial microbiome compared to healthy women (51.6% ± 38.3%).

Studies on the endometrial microbiome in RPL are scarce and varied in design. However, the findings are very similar to the available data from patients with RIF, most likely due to similar pathogenetic mechanisms disrupting early pregnancy: the absence of or reduction in lactobacilli and the presence of dysbiosis with pathogenic microbes and a wide variety of bacteria.

Given these findings, our study aimed to assess the presence and abundance of *Lactobacilli* in the endometrium in a large group of Bulgarian women with recurrent reproductive failure. In our retrospective cohort analysis of endometrial microbiomes, an inductive approach was used to assess the interim status without looking for an association with subsequent pregnancies. We separated the RIF and RPL groups into five subgroups according to *Lactobacillus* dominance, and correlation with age was performed.

## 2. Materials and Methods

### 2.1. Study Design and Population

We conducted a retrospective cohort analysis of endometrial samples from 199 women with RIF and RPL, who were diagnosed, treated and followed up at the CIRM Pleven (Clinical Institute for Reproductive Medicine) during the period from October 2019 to November 2022. All the included patients were Bulgarian females with an average age of 35.69 ± 5.16 years. An inductive approach was used to assess the interim status without looking for an association with subsequent episodes of RIF, RPL or live birth. The inclusion criteria were as follows: patients with RIF and RPL who provided written informed consent to participate in the study. The exclusion criteria were as follows: lack of written informed consent; untreated inflammatory processes of the vagina and cervix; present and untreated pathology of the adnexa and uterus; presence of diseases or medical conditions that threaten the patient’s health; and technical difficulties in performing an endometrial biopsy due to anatomical features.

### 2.2. Data Collection and Processing

All the endometrial biopsies were performed at MC CIRM Pleven by two physicians. The samples were processed at the MC CIRM Pleven laboratory utilizing real-time PCR (DNA Technology LLC; Moscow, Russia) following a prescribed protocol provided by the manufacturer. This ensured methodological consistency across the entire study period.

The methodology includes a quantitative assessment of total bacterial mass, including *Lactobacilli*. It is based on PCR amplification of targeted DNA sequences. The Femoflor 16 Real Time PCR Detection Kit is based on a fluorescent modification of the PCR method. Fluorescence intensity was measured at each cycle of reaction and analyzed with appropriate software (REAL-TIME PCR DETECTION KIT FEMOFLOR 16, Version 7, DNA-Technology). There is a PCR mix for human DNA (sample intake control (SIC)), which excludes pre-analytical error. In each analysis, the amount of material obtained from the uterine cavity is taken into account. If the amount of collected material is not sufficient to perform the analysis, it is necessary to repeat the sampling procedure. The method has an internal control (IC) to assess the quality of the polymerase chain reaction. Upon completion of the process, the software performs a relative quantitative analysis of total bacterial DNA, species-specific Lactobacilli DNA, and species-specific DNA of certain opportunistic pathogens. To exclude false-negative results, the amount of human DNA (SIC) is taken into account. Registration and interpretation of PCR results are made in automatic mode. The results include sample ID, test name, and the result of each test (quantity and a chart that allows relative comparison of normal flora and opportunistic pathogens in each sample). A qualitative pathogen analysis is also performed. The resulting graph is plotted against fluorescence intensity over the number of cycles for each tube.

### 2.3. Sample Collection Procedure

The endometrial biopsy was performed in the mid-luteal phase (21st–22nd day of a spontaneous menstrual cycle). All patients underwent a transvaginal ultrasound examination to determine the phase of the menstrual cycle. The patients were asymptomatic, with no evidence of current colpitis or endometritis. Endometrial samples were obtained from 103 patients (51.8%) with RIF (group 1) and 96 patients (48.2%) with RPL (group 2).

In the lithotomy position, after placing a sterile speculum, the vagina and cervix were thoroughly cleaned with 0.9% NaCl to remove as much vaginal contents and cervical mucus as possible. The speculum and vagina were not treated with disinfectants. The patient was restricted from using special hygiene procedures, vaginal douches, or topical medications or probiotic preparations. In case of antibiotic exposure, the procedure is postponed for at least 4 weeks. A flexible double-lumen “Intra-Uterine Insemination Catheter 180 mm” (Wallace^®^, Cooper Surgical, Inc., Shelton, CT, USA) was introduced into the uterine cavity. By applying negative pressure from a syringe with a volume of 1 mL mounted on the other end of the catheter and abrasive movement of the catheter in the uterine cavity, the uterine mucosa was abraded and aspirated. Before aspiration of the endometrial contents, the uterine cavity was not washed. Aspiration was terminated when the catheter came back to the level of the internal opening of the cervical canal. In order to prevent contamination with vaginal contents, contact between the catheter and vaginal walls was avoided. For the most correct interpretation of the obtained results, the sample needed to contain a large amount of endometrial mucosa. The assessment of the amount of the sample was made subjectively by the physician performing the sample collection, and in the case that the obtained material was deemed insufficient, a repeat biopsy was performed. The sample should contain a minimal amount of blood and mucus; this was ensured by carefully and tightly closing the test tube containing the obtained material. The sample was processed within one hour in order to preserve its integrity.

### 2.4. Statistical Analysis

Data were entered and processed with the statistical package IBM SPSS Statistics 25.0. and Excel on Office 2021. The significance level at which the null hypothesis is rejected was *p* < 0.05.

The following methods were applied: descriptive analysis, graphical analysis, Analysis of Variance, the Fisher–Freeman–Halton exact test, Fisher’s exact test and the χ2 test.

### 2.5. Ethical Considerations

This study was approved by the Research Ethics Committee of the Medical Center Clinical Institute for Reproductive Medicine Pleven (Approval No. 01/7 January 2019). Since it is an invasive procedure, every endometrial biopsy was performed after the patient willingly signed the informed consent form, which included comprehensive information about the procedure and consent for the use of their data for research. All measurements and procedures were conducted in accordance with the pertinent guidelines and regulations. This study did not involve any research products.

## 3. Results

### 3.1. Main Characteristics of Study Cohort

**Figure 1:**
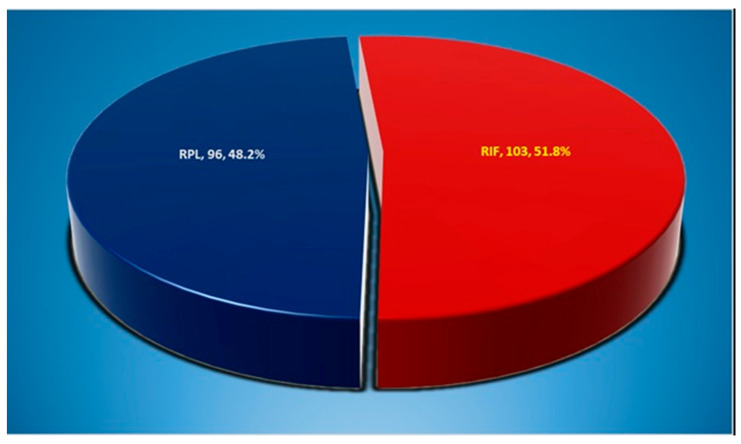
Distribution of the study cohort into the main study groups. The figure illustrates both the absolute number and relative percentage of patients in two primary groups: RIF (recurrent implantational failure), comprising 103 patients (51.8%) and RPL (recurrent pregnancy loss), comprising 94 patients (48.2%), demonstrating a nearly equal distribution between the groups with a slight predominance of RIF cases within the studied cohort.

A retrospective study was carried out on 199 patients of MC CIRM, Pleven, during the period of October 2019–November 2022. Two main groups were studied, designated RIF (recurrent implantational failure) (n = 103, 51.8%) and RPL (recurrent pregnancy loss) (*n* = 96, 48.2%) (Figure 1).

The study cohort had an average age of 35.69 ± 5.16 years, with a range of 24–54 years.

**Figure 2:**
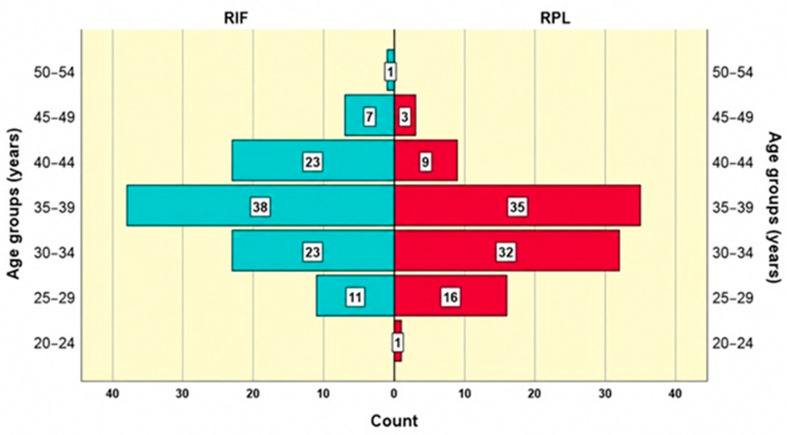
Distribution of the participants in the study by age and group. The figure presents the number of patients across different age categories for both RIF (recurrent implantation failure) and RPL (recurrent pregnancy loss) groups. The highest concentration of participants in both groups is observed in the 35–39 age range (RIF: 38; RPL: 35). A relatively higher number of RPL patients is seen in the younger age groups (25–34 years), whereas RIF cases are more prevalent in the older age groups (35 years and above). The extreme age categories (20–24 and 50–54 years) include only a minimal number of participants, indicating limited representation at the age margins.

The largest age group (38) in the RIF group was 35–39 years, followed by 30–34 and 40–44 years, with 23 each; there were no patients in the 20–24-year group. Among the women with RPL, the largest age group was 35–39 years (35), followed by 30–34 years with 32; there were no patients in the 50–54-year group. The median age was 37.0 (IQR 32.5–40.5) in the RIF group (*n* = 103) and 34.0 (IQR 31.0–38.0) in the RPL group (*n* = 96) with a statistically significant difference between the groups (*p* < 0.001, Mann–Whitney U test) (Figure 2).

**Figure 3:**
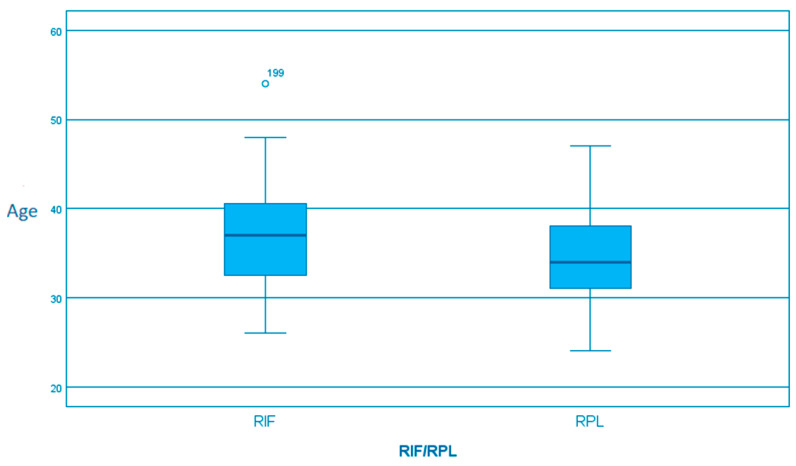
Boxplot of age distribution in the two study groups. The figure illustrates the distribution of age in the RIF (recurrent implantational failure) and RPL (recurrent pregnancy loss) groups, including median values, interquartile ranges, and variability. The RIF group demonstrates a higher median age compared to the RPL group (37.0 [32.5–40.5] vs. 34.0 [31.0–38.0] years, respectively). The difference between the groups is statistically significant (*p* < 0.001, Mann–Whitney U test), indicating the patients with RIF tend to be older than those with RPL.

The mean age was 36.91 ± 5.35 years in the RIF group and 34.39 ± 4.63 years in the RPL group (*p* = 0.0005); the women with RIF were, on average, 2.5 years older (Figure 3).

### 3.2. Composition of Endometrial Microbiome in Study Groups

In our study, five subgroups were defined based on *Lactobacillus* abundance: not detected, less than 30%, 30–50%, 51–85% and more than 85%.

**Figure 4:**
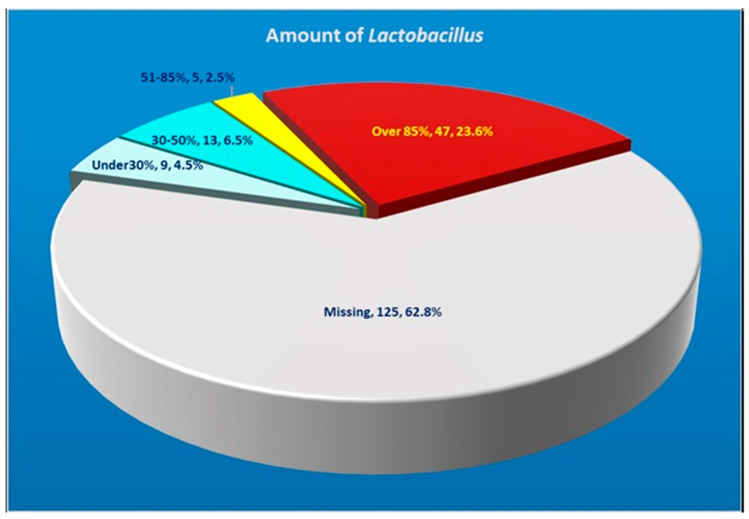
Distribution of female patients by *Lactobacillus* abundance. The chart illustrates the proportion of patients across predefined subgroups based on *Lactobacillus* levels. The majority of patients (62.8%) showed no detectable *Lactobacillus* presence, indicating a substantial deviation from normal microbiome composition. A considerable proportion (23.6%) exhibited high *Lactobacillus* abundance (>85%), while smaller fractions were distributed among intermediate levels: 30–50% (6.5%), <30% (4.5%), and 51–85% (2.5%). These findings highlight the predominance of dysbiotic conditions within the studied population.

A normal concentration of *Lactobacilli*, according to the current data, was only found in a quarter of the cases. In the remaining 76.4%, there is a significant disturbance in the normal composition of the microbiome. The *Lactobacillus* abundance results are shown in Figure 4.

The largest subgroup (62.8%) consisted of patients with no detectable presence of *Lactobacilli*, followed by those with levels above 85% (47 or 23.6%). The smallest subgroup (2.5%) consisted of those with 51–85% *Lactobacilli*. In both groups, 13.5% of patients showed a low *Lactobacillis* abundance.

**Figure 5:**
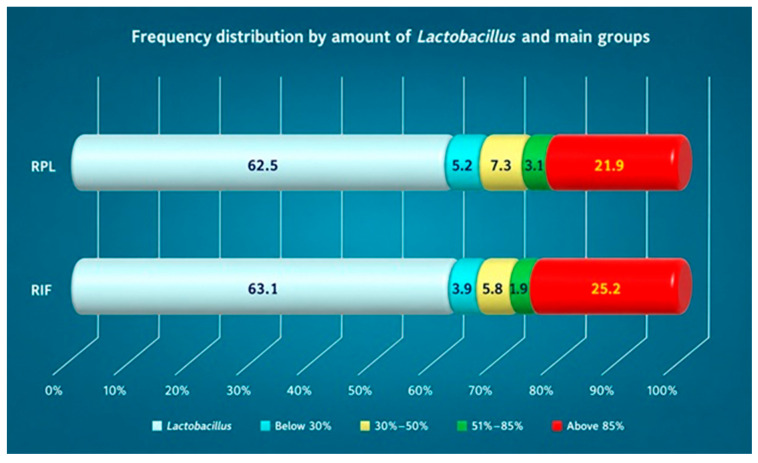
Distribution of female patients by *Lactobacillus* abundance and main group. The figure presents the comparative frequency distribution of *Lactobacillus* abundance across the RPL and RIF groups. In both groups, the majority of patients exhibited no detectable Lactobacillus (62.5% in RPL and 63.1% in RIF), indicating a high prevalence of microbiome imbalance. A notable proportion of patients showed high *Lactobacillus* abundance (>85%) with a slightly higher percentage in the RIF group (25.7%) compared to the RPL group (21.9%). The intermediate abundance categories (under 30%, 30–50%, and 51–80%) were represented by relatively small fractions in both groups, with consistently lower proportions observed in the RIF group. Overall, the distribution patterns between the two groups are similar, with minor differences in higher *Lactobacillus* levels.

We also compared the amounts of *Lactobacilli* in the two groups. There was no statistically significant relationship between *Lactobacillus* abundance and belonging to the RIF or RPL group (Table 1 and Figure 5). These data may suggest a common pathogenetic mechanism of damage to the endometrial environment, which affects implantation and early pregnancy development.

### 3.3. Age-Related Changes in Lactobacillus Abundance

**Figure 6:**
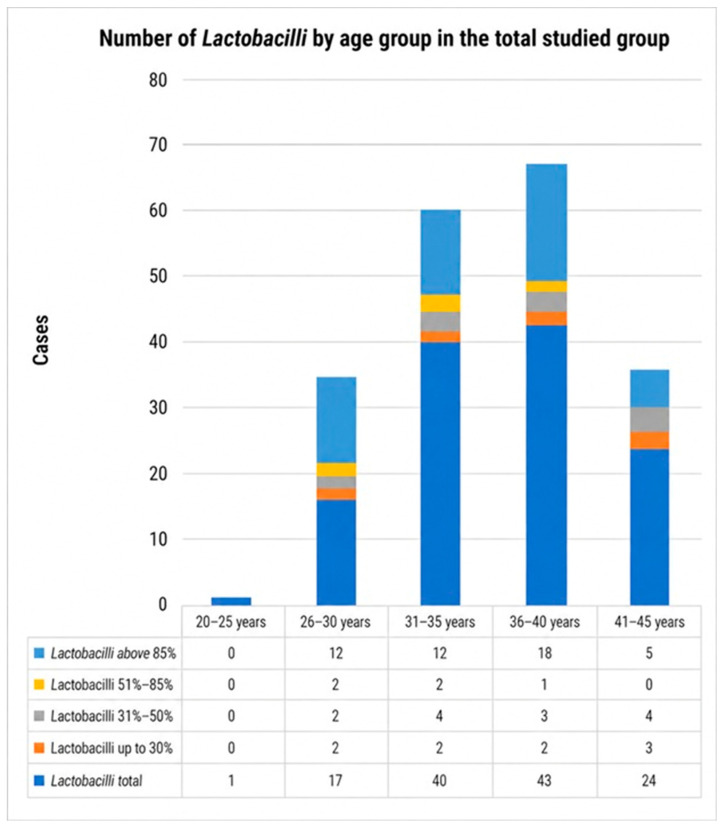
Distribution of *Lactobacillus* abundance across age groups in the studied population. Overall, the data suggest an age-related shift toward reduced *Lactobacillus* dominance and increased microbiome imbalance.

**Figure 7:**
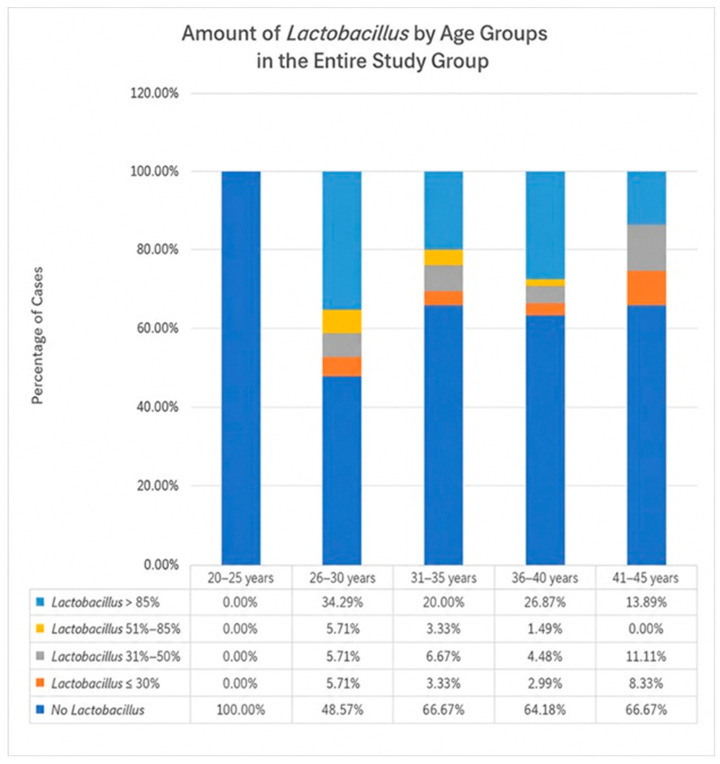
The chart illustrates the distribution of *Lactobacilli* levels across different age groups within the studied population. A notable trend is observed in the youngest group (20–25 years), where all cases (100%) show a complete lack of *Lactobacilli*. In contrast, older age groups demonstrate a more heterogeneous distribution. Overall, the data suggest that while the absence of *Lactobacilli* is predominant across most age groups, especially in younger and older extremes, intermediate and higher levels become more variable with increasing age.

The results obtained for the entire group confirmed the negative effect of age on *Lactobacillus* saturation and dominance. The age group with the highest percentage of patients with undetectable *Lactobacillus* levels was the 41–45-year group (66.67%), and the one with the lowest percentage was the 26–30-year group (48.57%). This latter group had the highest percentage of patients with a *Lactobacillus*-dominated microbiome—34.29% vs. 13.89% in the 41–45-year group (Figure 6 and Figure 7).

**Figure 8:**
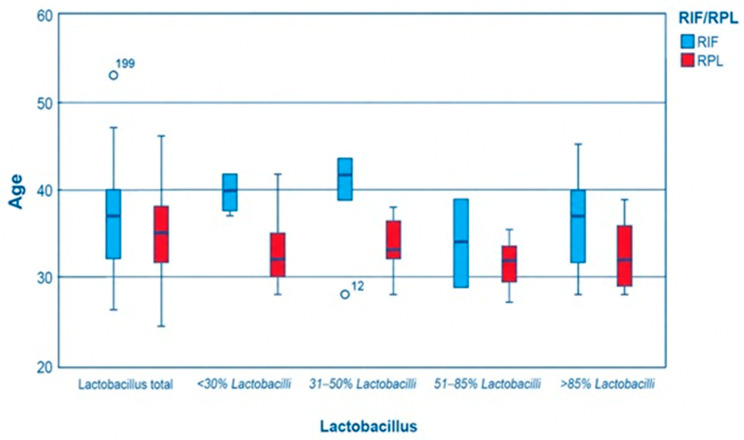
Boxplot representation of age distribution stratified by *Lactobacillus* abundance in patients with recurrent implantation failure (RIF) and recurrent pregnancy loss (RPL). Participants were categorized into five groups based on the relative abundance of *Lactobacillus* (missing, <30%, 31–50%, 51–85%, and >85%). Each boxplot shows the median (central line), interquartile range (IQR; 25th–75th percentile, box), whiskers extending to 1.5 × IQR, and outliers plotted as individual points. Across all categories, the RIF group (blue) generally demonstrates a slightly higher median age compared to the RPL group (red), although there is substantial overlap in IQRs, indicating similar variability between groups. No consistent trend in age distribution is observed with increasing *Lactobacillus* abundance, and overall, the distributions suggest no significant differences in age between RIF and RPL patients across the defined categories.

A statistically significant difference in mean age between the RIF and RPL groups was found among those with 31–50% and greater than 85% *Lactobacilli* in their endometrium, i.e., among the patients with 31–50% *Lactobacilli*, the mean age was 39.8 ± 6 years for those with RIF and 33.7 ± 3.5 years for those with RPL (*p* = 0.046). Among the patients with a normal *Lactobacillus* abundance (greater than 85%), the mean age was 36.4 ± 4.8 years among those with RIF and 32.5 ± 3.5 years among those with RPL (*p* = 0.004) (Figure 8).

No statistical significance was found in the distributions of RIF and RPL patients with respect to *Lactobacilli* abundance group within the different age groups.

**Figure 9:**
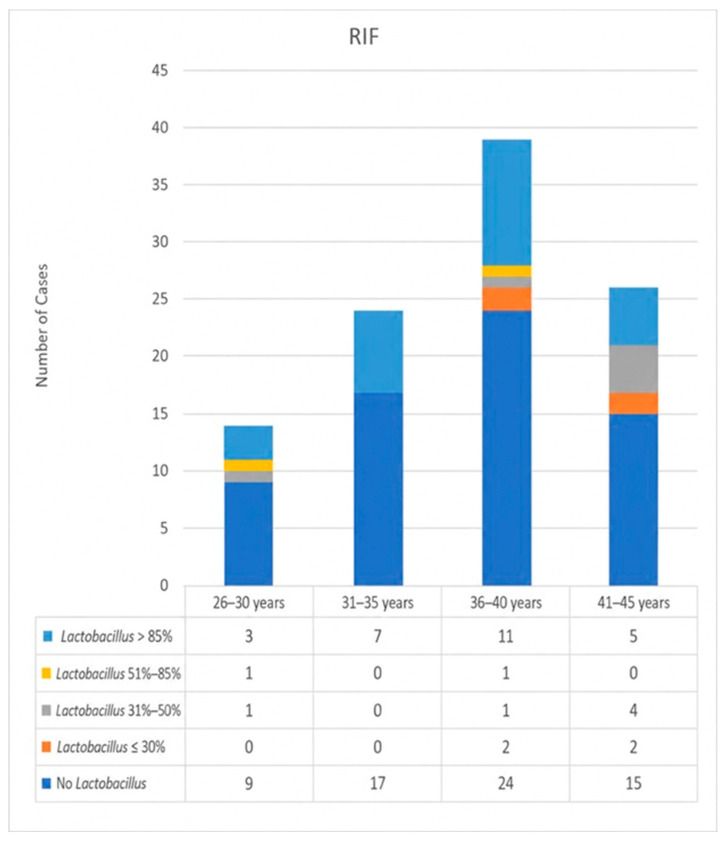
Distribution of *Lactobacillus* abundance across age groups in patients with recurrent implantation failure (RIF). The stacked bar chart presents the number of cases within four age categories, stratified by levels of *Lactobacillus* abundance. The largest number of cases is observed in the 36–40-year group, followed by 41–45 and 31–35 years, while the 26–30-year group shows the lowest frequency. Across all age groups, cases with low or absent *Lactobacillus* predominate, whereas higher *Lactobacillus* abundance (>85%) is less frequent. Overall, the data indicate that non-*Lactobacillus*-dominant microbiome profiles are more common in RIF patients regardless of age.

**Figure 10:**
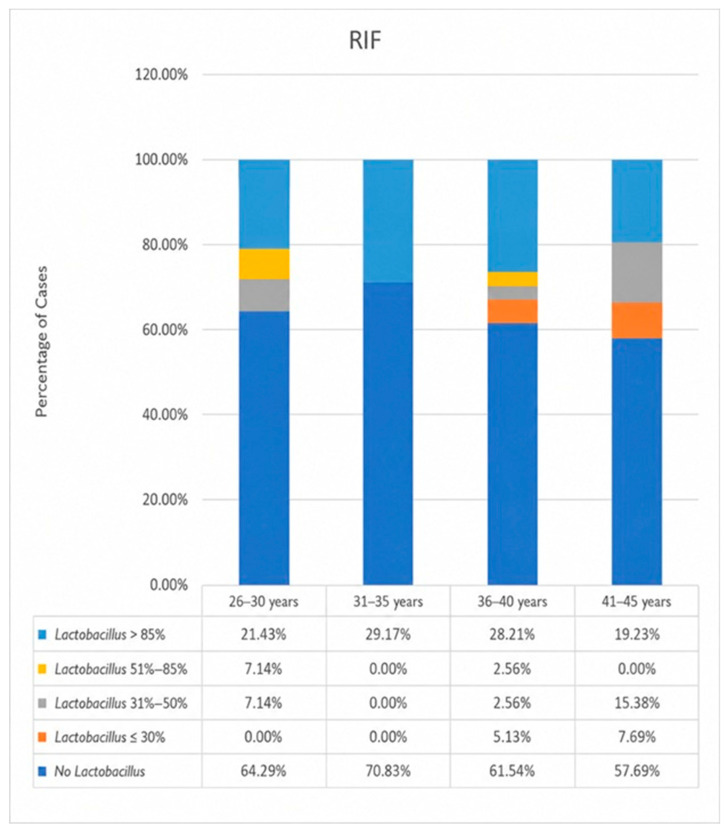
Percentage distribution of *Lactobacillus* abundance across age groups in patients with recurrent implantation failure (RIF). The stacked bar chart presents the relative proportions of *Lactobacillus* categories within four age groups, with each column summing to 100%. Across all age categories, lack of *Lactobacillus* constitutes the largest proportion, particularly in the 31–35-year group, indicating a predominance of non-*Lactobacillus*-dominant microbiome profiles in RIF patients. Higher *Lactobacillus* abundance (>85%) is relatively more frequent in younger and middle-aged groups but shows a slight decrease in the 41–45-year group. Intermediate abundance levels (31–50% and 51–85%) remain generally low across all categories. Overall, no clear age-dependent trend toward increasing *Lactobacillus* dominance is observed, and the data suggest a consistent predominance of low or absent *Lactobacillus* across all age groups.

In the RIF group, the age group with the highest percentage of patients with a *Lactobacillus*-dominant microbiome was the 31–35-year group (29.17%); this age group also had the highest percentage of patients with an undetectable *Lactobacillus* abundance (70.83%). The other age groups showed different degrees of *Lactobacillus* reductions (Figure 9 and Figure 10).

**Figure 11:**
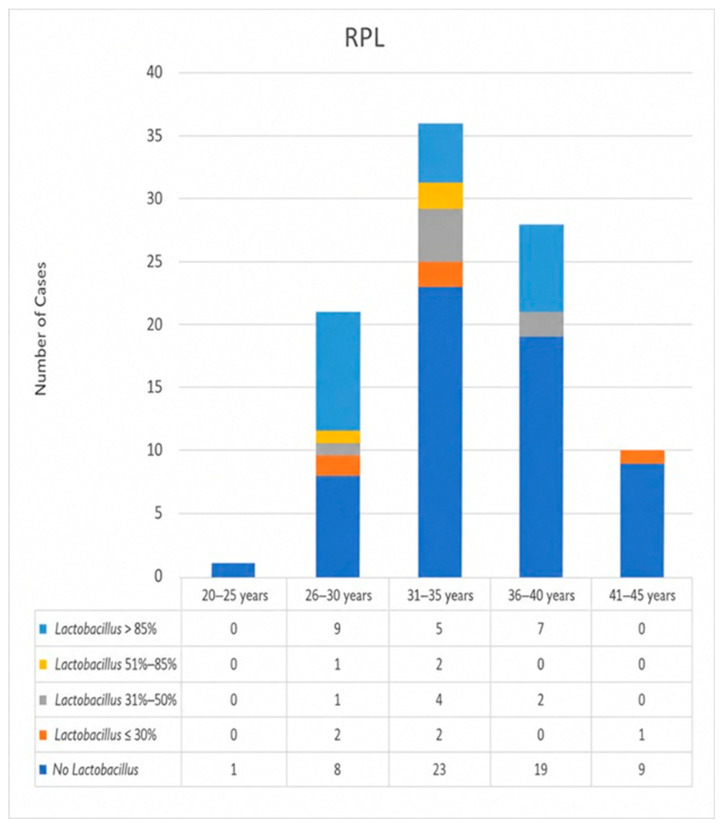
Distribution of *Lactobacillus* abundance across age groups in patients with recurrent pregnancy loss (RPL). The stacked bar chart presents the number of cases within five age categories, stratified by levels of *Lactobacillus* abundance. The highest number of cases is observed in the 31–35-year group, while the 20–25-year group shows minimal representation. Across all age groups, lack of *Lactobacillus* constitutes the largest proportion of cases, indicating a predominance of non-*Lactobacillus*-dominant microbiome profiles in RPL patients. Higher *Lactobacillus* abundance (>85%) is present but less frequent and varies across age groups. No clear age-related trend in *Lactobacillus* dominance is evident, and the overall distribution suggests that reduced or absent *Lactobacillus* is common across all age categories in the RPL group.

**Figure 12:**
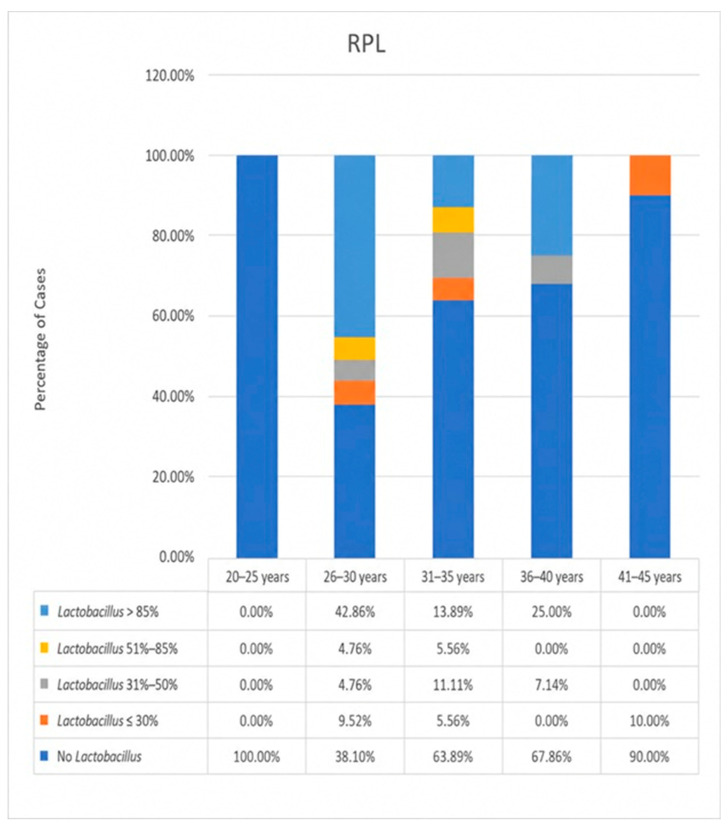
Percentage distribution of *Lactobacillus* abundance across age groups in patients with recurrent pregnancy loss (RPL). The stacked bar chart illustrates the relative proportions of *Lactobacillus* abundance categories within five age groups, with each column totaling 100%. Across most age groups, the lack of *Lactobacillus* represents the predominant proportion. Higher *Lactobacillus* abundance (>85%) is more prominent in the 26–30 and 36–40 age groups, but remains variable overall. Overall, the distribution does not demonstrate a clear trend of increasing *Lactobacillus* dominance with age, and the findings suggest that reduced or absent *Lactobacillus* is common across all age groups in RPL patients.

In the RPL group, the age group with the highest percentage of patients with a *Lactobacillus*-dominant microbiome was 26–30 years (42.86%); the 41–45-year group had the lowest percentage (0%). Accordingly, this latter age group had the highest percentage of patients with an undetectable *Lactobacillus* abundance (90%), while the lowest percentage was observed in the 26–30-year group (38.1%) (Figure 11 and Figure 12).

## 4. Discussion

Reproductive failures may be fundamentally related to changes in the microbiome of the reproductive tract. Data in recent years have focused on the microbiome of the lower reproductive tract, while the microbiome of the upper reproductive tract has been relatively poorly studied. The initial research on the endometrial microbiome was a major breakthrough and broke the stereotype of uterine sterility. Initially, this research strongly supported *Lactobacillus* dominance as a critical component of uterine eubiosis before it shifted to the idea that reduced *Lactobacillus* abundance is the condition for successful pregnancy. Thus, there is still no clear definition and unified opinion on endometrial eubiosis, although all this research does agree that uterine sterility is not a major cause of reproductive failures.

Our analysis revealed a striking depletion of *Lactobacilli* in the endometrial microbiome of patients with reproductive failure. These results are in accordance with the current opinion that uterine eubiosis is a requirement for successful implantation and early pregnancy development. According to Moreno et al. [8], in fertile women, the endometrial microbiome is dominated by *Lactobacilli.* However, our results from patients with reproductive failure from the Bulgarian ethnic group contradict those of Moreno et al. in fertile women. There was a high frequency of non-*Lactobacillus*-dominated endometrial microbiomes in our patients (76.4%), with only 23.6% having a normal *Lactobacillus* abundance according to the criteria of Moreno et al. Moreover, the frequencies were similar in the two studied groups (RIF and RPL). These conflicting results can be explained by the strict selection criteria and preparation of the patients for the study. Another possible influencing factor that should not be overlooked is the homogenous ethnic background of the female patients—all of them are from the Bulgarian ethnic group. This high frequency of non-*Lactobacillus*-dominated endometrial microbiomes in our group also corresponds to a high frequency of reproductive failures, and there could be missed diagnoses that require etiological treatment. These results cannot be generalized to women from other ethnic groups. Information on ethnic variations in the composition of endometrial microbiomes is scarce and is based mainly on data on variations in the vaginal microbiome [11]. These ethnic variations in vaginal microbiome may contribute to uterine variations due to uterine peristalsis, which can introduce bacteria from the vagina [12]. Our study does not include a control group of healthy Bulgarian women with normal reproductive histories, which is a limitation of the study. We emphasize that microbiome composition may vary depending on population characteristics, diet and geographic factors [1,8]. We cannot fully determine whether the observed findings represent the local population or are specifically associated with reproductive failure. This limitation is a subject of further studies and research.

These results are comparable to the results of Kitaya et al. [13]. They concluded that among the infertile population, especially IVF patients, a *Lactobacillus*-dominated microbiome and a statistically significantly lower percentage of *Lactobacilli* were less common.

The hypothesis that a *Lactobacillus*-dominated endometrial microbiome is required for implantation and early pregnancy development is supported by the findings of Kyono et al. [14]. They found that after embryo transfer, pregnancy occurred in 61.3% of those with a *Lactobacillus* abundance above 80% and in 40% of those with an abundance below 80%. These authors did not claim a clear benefit of a *Lactobacillus*-dominated endometrial microbiome for pregnancy outcomes but rather emphasized that restoring *Lactobacillus* dominance could benefit implantation.

This is also the opinion of Keburiya’s team [9], who found that 89.2% of patients with RIF showed microflora with a dominance of *Lactobacilli*. According to them, there is no clear evidence that the presence of a *Lactobacillus*-dominated endometrial microbiome has a beneficial effect on the occurrence and outcomes of pregnancy, but its restoration may have a positive effect on implantation and early pregnancy. They found no effect of opportunistic bacteria on pregnancy rates. Most likely, all these effects are due to the fact that the uterine microbiota is a collection of functionally associated microorganisms [15]. Certain microorganisms in the uterine cavity are involved in maintaining homeostasis. They form biofilms comprising bacterial communities that play important roles and have certain physiological properties. Normal biofilms in the human body include those formed by the physiological microflora of the skin, oral cavity, vagina and intestines. The formation of pathological biofilms is associated with chronic inflammatory processes.

Regarding the RIF group, our results are consistent with those of Ichiyama et al. [10], who found that, among 145 RIF patients, 51.2 ± 37.5% showed an absence of *Lactobacilli*. Their study included a control group of healthy women, where 28.6% showed a presence of *Lactobacilli* while 51.6 ± 38.3% showed an absence, i.e., there was no statistically significant difference between the two groups. For this reason, these authors do not accept the concept of a *Lactobacillus*-dominated microbiome as a biomarker for implantation success. A similar opinion was expressed by the team of Kyono [14] in their study. According to their data, there was no difference in the outcome of IVF procedures in patients with a *Lactobacillus*-dominated vs. non-*Lactobacillus*-dominated microbiome. Therefore, they believe that it is necessary to revise the reference limits for *Lactobacilli* in fertile women in cases where the presence and abundance of *Lactobacilli* are used as a biomarker for implantation failure. According to Lozano et al. [16], in patients with RIF, there was a lower abundance of *Lactobacilli*. The absence of or reduction in Lactobacilli in these cases most likely creates favorable conditions for the movement of microorganisms into the uterine cavity and a subsequent negative effect on the occurrence and development of a normal pregnancy, which suggests that a microbiome with a low biomass could allow pathogens to enter.

Studies on endometrial microbiomes in RPL are scarce and vary, but the findings are very similar to the available data from patients with RIF, most likely due to both conditions being associated with an absence of or reduction in *Lactobacilli* and the presence of dysbiosis with pathogenic microbes and a wide variety of bacteria. In our RPL group, *Lactobacilli* were absent in 62.5% of cases and were abundant in 21.9%. Barinova et al. [17] studied the endometrial microbiome in healthy fertile women and in women with RPL. In the RPL group, *Lactobacilli* were the most abundant in 30.3% compared to 29.4% in the group of healthy fertile women. There was no statistically significant difference between the two groups. These results align with the concept of *Lactobacillus* dominance; however, this dominance is below the accepted 90% threshold, so they cannot prove a negative effect of a reduced *Lactobacilli* abundance on reproductive outcomes.

Age is a well-known factor associated with decreases in *Lactobacillus* abundance and increases in microbial diversity in the endometrial microbiome, leading to the development of dysbiosis. Our results are similar to those in the published literature. In 2023, Fujii’s group [18] investigated the relationship between age and endometrial receptivity and endometrial *Lactobacillus* level. They found a shifted implantation window in advanced age. Since advanced age is accompanied by a decrease in *Lactobacilli*, they believe that *Lactobacillus* abundance and their interaction with endometrial host cells are responsible for the development of uterine receptivity and preparing the uterus for implantation.

Wang et al. [6] conducted a broader analysis of age-related *Lactobacillus* changes and found that the levels are stable until about 40 years of age, after which, they begin to fluctuate, and in the period of 40 + to 60 years, they significantly decline. This study was conducted in the follicular phase of the menstrual cycle and included a control group of healthy women.

Odawara et al. [7] investigated factors influencing the intrauterine microbiota in Japanese women. They found a significant difference in terms of age and parity. In women over 36 years of age, there was a significant decrease in intrauterine *Lactobacilli*, especially in parous compared with non-parous women. The most likely reasons for this are that the broken integrity of the cervix at birth and postpartum amenorrhea with low estrogen levels allow for the invasion and colonization of the uterine cavity with bacteria other than *Lactobacilli*. In our research, we did not analyze the influence of previous births, abortions, curettages and other intrauterine manipulations, including embryo transfer and intrauterine insemination, which could have breached the natural barrier and allowed the invasion of microorganisms from the vagina.

Interesting results were obtained from a study conducted during ovarian stimulation during in vitro fertilization (IVF) cycles. In this study, women >35 years displayed a greater increase in *Lactobacillus* numbers compared to younger women based on samples from the tip of the embryo catheter at embryo transfer [19]. Estrogen levels affect *Lactobacillus* levels in the human vagina, where the relative abundance of *Lactobacilli* is >70%; in other mammals, *Lactobacilli* rarely comprise more than 1% [20]. According to Zukic et al. [19], variation in *Lactobacillus* levels is more pronounced in older women during ovarian stimulation.

The functional effects of *Lactobacilli* in the endometrium are not directly proportional to their abundance [21]. These relationships are complex and may not follow a strict linear dose–response pattern [22]. Small changes do not always lead to small effects. The concept of threshold is accepted [8], and *Lactobacillus* levels above 80–90% are considered dominant. A certain level of *Lactobacilli* is needed to keep the uterine environment stable and protective, and even a small decrease can cause dysbiosis [23]. According to this hypothesis, *Lactobacillus* dominance is more important than small changes in its amount.

## 5. Conclusions

This study highlights the importance of uterine microbiome evaluation in women with reproductive failure. Although more data are needed to establish the exact role of uterine *Lactobacilli* in embryo implantation and early pregnancy development, they must be considered in the diagnosis, counseling and treatment of these women. Age-related depletion in *Lactobacilli* abundance may play a significant role in worsening IVF outcomes and increasing miscarriages. Improving our knowledge could lead to better pregnancy outcomes and live birth rates.

## Figures and Tables

**Table 1:** Distribution of all patients and RIF and RPL groups by *Lactobacillus* abundance (*p* = 0.926).

*Lactobacillus* Abundance	** **	Total	RIF	RPL
Not Detected	n	125	65	60
	%	62.8	63.1	62.5
Under 30%	n	9	4	5
	%	4.5	3.9	5.2
30–50%	n	13	6	7
	%	6.5	5.8	7.3
51–85%	n	5	2	3
	%	2.5	1.9	3.1
Over 85%	n	47	26	21
	%	23.6	25.2	21.9
Total	n	199	103	96
	%	100.0	100.0	100.0

## Data Availability

The datasets used and data analyzed during the current study will be made available upon reasonable request to the corresponding author.

## Abbreviations

The following abbreviations are used in this manuscript:

RIF	Recurrent implantation failure
RPL	Recurrent pregnancy loss
PCR	Polymerase chain reaction
